# Seasonal Evaluation of Phlorotannin-Enriched Extracts from Brown Macroalgae *Fucus spiralis*

**DOI:** 10.3390/molecules26144287

**Published:** 2021-07-15

**Authors:** Belén Almeida, Sónia Barroso, Ana S. D. Ferreira, Pedro Adão, Susana Mendes, Maria M. Gil

**Affiliations:** 1 MARE—Marine and Environmental Sciences Centre, Polytechnic of Leiria, Cetemares, 2520-620 Peniche, Portugal; 4180293@my.ipleiria.pt (B.A.); pedro.adao@ipleiria.pt (P.A.); 2 Associate Laboratory i4HB—Institute for Health and Bioeconomy, School of Science and Technology, NOVA University Lisbon, 2819-516 Caparica, Portugal; asd.ferreira@fct.unl.pt; 3 UCIBIO—Applied Molecular Biosciences Unit, Department of Chemistry, School of Science and Technology, NOVA University Lisbon, 2819-516 Caparica, Portugal; 4 MARE—Marine and Environmental Sciences Centre, ESTM, Polytechnic of Leiria, Cetemares, 2520-620 Peniche, Portugal; susana.mendes@ipleiria.pt (S.M.); maria.m.gil@ipleiria.pt (M.M.G.)

**Keywords:** *Fucus spiralis*, antioxidant activity, polyphenols, phlorotannins

## Abstract

*Fucus spiralis* that was collected in the four seasons was submitted to an extraction with ethanol:water (crude extracts Et80), followed by a liquid–liquid fractionation with organic solvents (fraction He from n-hexane; aqueous fractions AQ1, AQ2, AQ3 and AQ4; ethyl acetate fraction EA), with the aim of obtaining phlorotannin-enriched extracts. All the extracts (Et80, He, AQ1, AQ2, AQ3, AQ4 and EA) that were obtained for the *F. spiralis* of the four seasons were evaluated for their antioxidant capacity and total phenolic compounds. The summer extracts presented the highest contents in polyphenols (TPC), as well as the highest ferric reducing antioxidant power (FRAP), when compared to the samples from the other seasons. The reductive percentage of the DPPH (2,2-diphenyl-1-picryl-hydrazyl-hydrate) compound was similar between the seasons. For all the seasons, the EA extract showed the highest polyphenol content (TPC), and the highest antioxidant capacity (highest ferric reducing power (FRAP) and lowest concentration needed to reduce 50% of the DPPH compound), which is in agreement with a phlorotannin-enriched fraction. This study revealed that the polyphenol content and antioxidant power of the *F. spiralis* extracts are influenced by the time of harvest, as well as by the solvents used for their extraction.

## 1. Introduction

*Fucus* is an abundant and widely distributed genus of brown, perennial and edible macroalgae that inhabits the cold-temperate water regions along the littoral and sublittoral rocky shorelines of the Northern Hemisphere. However, the population is dependent on the season, solar exposure, salinity, and geographical origin. Since they inhabit intertidal zones, which is an environment of rapidly changing physical conditions due to the turning tides, they need to develop a series of metabolites that will help them endure this highly competitive and aggressive environment. This leads to the production of quite specific and potent bioactive molecules, which may be a valuable resource as nutritional or pharmaceutical supplements [1].

**Figure 1:**
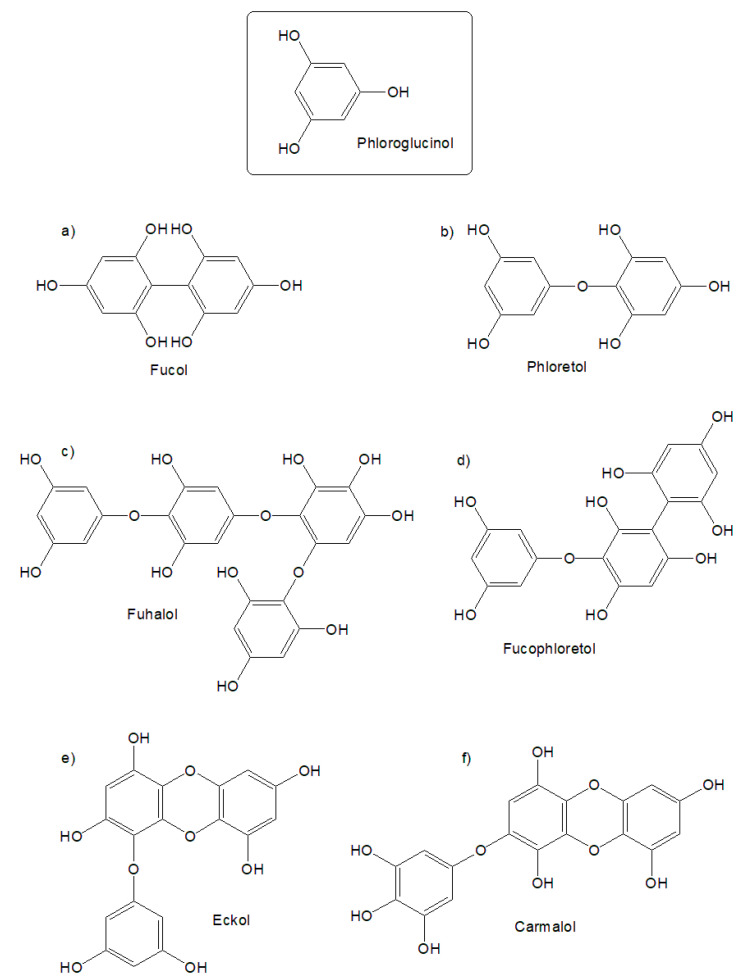
Examples of phlorotannins present in brown algae. Depending on inter-phloroglucinol linkage, phlorotannins are divided into (**a**) fucols (phenyl linkage), (**b**) phloretols (ether linkage), (**c**) fuhalols (ether linkage and one or more additional hydroxyl group), (**d**) fucophloretols (ether and phenyl linkage), and (**e**) eckols and (**f**) carmalols (dibenzoidin linkage).

One of the main features of brown algae is the presence of many phenolic derivatives, such as phlorotannins [2]. These compounds are polyphenols that are derived from phloroglucinol, and are mostly present in the orders Fucales and Laminariales. These polyphenols are divided into the following five groups depending on inter-phloroglucinol linkage: fucols (Figure 1a; phenyl linkage), phloretols (Figure 1b; ether linkage), fuhalols (Figure 1c; ether linkage and one or more additional hydroxyl group), fucophloretols (Figure 1c; ether and phenyl linkage), and eckols and carmalols (Figure 1e,f; dibenzoidin linkage). The content of phlorotannins in brown algae reaches 15% of the dry weight, depending on the habitat of the algae, the time of their sampling, the intensity of illumination, and other factors [3]. Phlorotannins have been shown to have potent antioxidant and anti-inflammatory activities [2], anti-viral activity [4], anti-coagulant, anti-diabetic, anti-cancer activities, as well as enzyme-inhibiting properties [5]. This is due to their chemical structure, with up to eight interconnected rings, or phloroglucinol units (Figure 1), which effectively prevent oxidation, due to the higher number of functional phenolic groups [6].

Polyphenolic compounds have become common constituents of the human diet, due to their therapeutic properties (antioxidative, antibacterial, anti-inflammatory, among others). Indeed, several studies have described the antioxidant and nutritional potential of brown algae, and their application in commercial products [7,8]. Brown algae belonging to the Fucus genus, including *F. vesiculosus* and *F. spiralis*, are rich in bioactive polyphenolic secondary metabolites, such as phlorotannins, and other compounds such as carotenoids, tocopherols, sulphated polysaccharides, peptides, and polyphenols, all of which exhibit antioxidant activity [9,10]. Those with antioxidant activity, namely, phenolic compounds, inhibit lipid oxidation in food products, by transforming free radicals into non-radicals by donating electrons and hydrogen molecules or by chelating transition metals [11]. Although the use of phlorotannins’ extracts in the formulation of functional foods is limited, due to the difficulty in their isolation or production at an industrial scale, they have shown to be strong bioactive agents that have health-promoting qualities when applied to food products [6,12,13]. The use of phlorotannin-enriched extracts as active food package material has also been developed to effectively preserve the quality of food products and extend their shelf-life, without the need for artificial antioxidants [14,15,16].

For the extraction of the compounds of interest from macroalgae, conventional solvent extraction methods, such as Soxhlet, maceration, hydrodistillation, can be used. Alternative methods, such as microwave-assisted extraction (MAE), enzyme-assisted extraction (EAE), and ultrasound-assisted extraction (UAE), are often classified as green methods due to the benefits they present over conventional methods, by reducing the time needed for extraction and the amount of solvent used, as well as avoiding the use of non-ecological solvents [17]. Solvent extraction is the most common method used for the extraction of Phlorotannins; these are extracted from fresh macroalgae or lyophilized material using ethanol or acetone, or their aqueous solutions. When the extraction is carried out for subsequent use in the food, pharmaceutical or cosmetic industries, ethanol aqueous mixtures are preferred [18]. Subsequently, the fractionation of the alcoholic or aqueous extract by liquid–liquid extraction with organic solvents, often ethyl acetate, yields an extract that is rich in phlorotannins [3]. Alternative methods have been used to extract phlorotannins from brown algae. MAE has been used to selectively extract phlorotannins from *Fucus vesiculosus* using hydroethanolic solvents, affording recovery profiles and yields of phlorotannins from the macroalgae that are similar to conventional extraction methods [19]. EAE was used to extract these phenolic compounds from *Sargassum* spp., while comparing this method to the widely used conventional solid–liquid extraction. The study revealed that by using enzymes, the efficiency of the extraction increased, which, in turn, improved the bioactivity, quality and quantity of the extracts [8]. UAE was employed to extract phlorotannins and polysaccharides from *Silvetia compressa*, significantly enhancing their extraction, using only hydroethanolic solvents [20]. Other alternative methods have been tested to improve the amount of phlorotannins that are extracted from algae material, such as subcritical water hydrolysis, centrifugal partition extraction (CPE), supercritical fluid extraction (SFE), and pressurized liquid extraction (PLE), but the high cost of these methods and the low availability reduces the possibility to apply them [21].

The aim of this work was the isolation of phlorotannin-enriched extracts from *F. spiralis*, for application as natural antioxidants in food products, using a simple extraction method and low-toxicity solvents. A seasonality study was also conducted in order to acknowledge the best time of the year for the algae collection.

## 2. Results and Discussion

### 2.1. Extraction and Characterization

**Figure 2:**
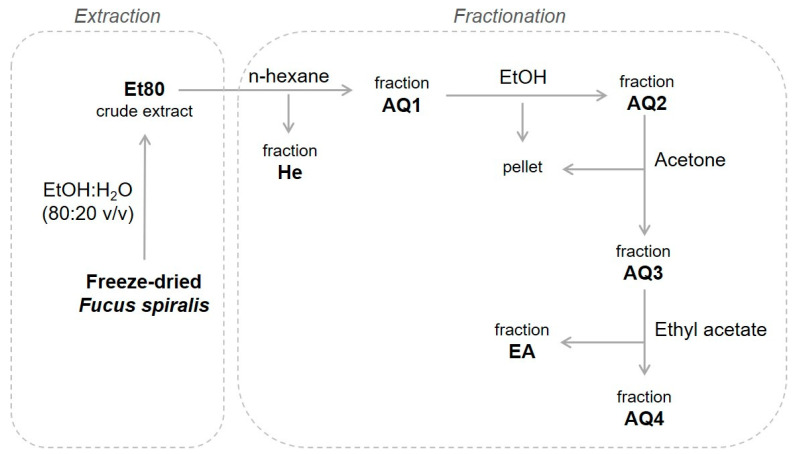
Process for the extraction and fractionation of *F. spiralis*.

Figure 2 shows the process that was followed for the extraction and fractionation of *F. spiralis* from the four seasons. The biomass was first extracted with 80% ethanol, and the crude ethanolic extracts (Et80) were then fractionated through a liquid–liquid extraction procedure, using organic solvents with different polarities. The fractionation procedure is intended to remove lipids, pigments, polysaccharides, and proteins/amino acids, yielding ethyl acetate extracts that are enriched in phlorotannins. The original protocol [21] involved an extraction step with dichloromethane, which was skipped in order to achieve a less toxic extraction process that would be compatible with applications in the food industry.

#### 2.1.1. Total Phenolic Content (TPC)

**Figure 3:**
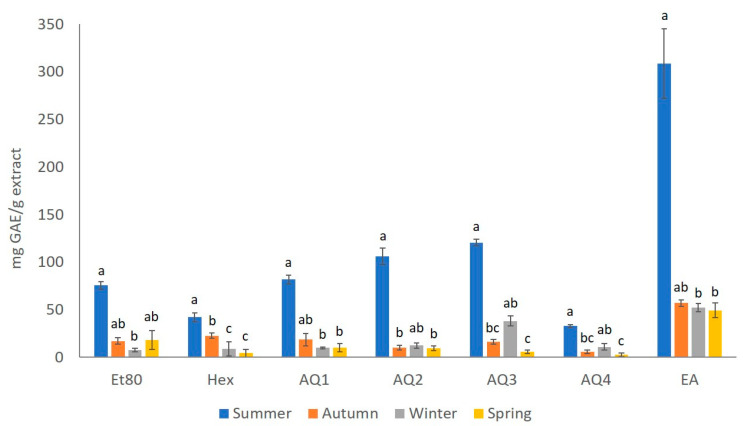
Total phenolic content (mg GAE/g extract) of the extracts obtained from *F. spiralis* collected in the four seasons. Results are represented as mean ± SD (*n* = 4) and different lowercase letters represent significant differences (Dunn test, *p*-value ≤ 0.05) between seasons for each extract.

The phenolic content (TPC) of the extracts that were obtained from *F. spiralis* was estimated using the Folin–Ciocalteu test, and the results are presented in Figure 3. A significant difference was observed, not only between the extracts, but also between the seasons. The extract that showed the highest amount of phenols in every season was EA, having 308.63 mg GAE/g of extract for the summer season, 56.78 mg GAE/g of extract for the autumn season, 52.28 mg GAE/g of extract for the winter season, and 49.17 mg GAE/g of extract for the spring season. For the summer, autumn and spring seasons, the extract with the lowest phenolic content is AQ4, with 32.65, 5.60, and 2.49 mg GAE/g of extract, respectively; while the Et80 extract from the winter season has seemingly the lowest amount of phenolic content, with 7.33 mg GAE/g of extract.

Overall, the extracts with the higher amounts of phenols are from the summer season, with a TPC that ranges between 32.646 mg GAE/g of AQ4 extract and 308.634 mg GAE/g of EA extract. On the other hand, the results indicate that the extracts with the lowest amount of phenols are from the spring season, with a TPC ranging from 2.49 mg GAE/g of the AQ4 sample and 49.170 mg GAE/g of the EA sample.

The results that were obtained for the effect of the season on the TPC values, showed statistically significant differences (Kruskal–Wallis, $\chi^{2}$= 54.95, *p*-value ≤ 0.01; Figure 3). Therefore, it was possible to observe that the extracts from the summer season showed a significant difference from the extracts of most of the other seasons, regardless of the solvent used in the extraction (Dunn test, *p*-value ≤ 0.05; Figure 3).

When analyzing the effect of the seasons over the TPC yield of the Et80 extracts, a statistical difference could be seen (Kruskal–Wallis, $\chi^{2}$ = 12.23, *p*-value ≤ 0.01; Figure 3). It was possible to observe that the extracts with higher TPC values were from the summer samples, with values that were significantly higher than those observed for the winter samples, which was the season with extracts presenting lower TPC values (Dunn test, *p*-value ≤ 0.05). The effect of the seasons on the TPC yield of He extracts also showed a statistical difference (Kruskal–Wallis, $\chi^{2}$ = 11.50, *p*-value ≤ 0.01; Figure 3). The He extract with the highest TPC value was from summer, followed by He from autumn, both of which were significantly higher than the He extracts from spring and winter, which presented lower TPC yields (Dunn test, *p*-value ≤ 0.05; Figure 3). The AQ1 extracts also presented a seasonal effect on the TPC yields (Kruskal–Wallis, $\chi^{2}$ = 12.74, *p*-value ≤ 0.01; Figure 3). The AQ1 extract from summer showed the highest TPC value, which was significantly higher than those obtained for the AQ1 extracts from winter and spring (Dunn test, *p*-value ≤ 0.05; Figure 3). The statistical difference in the effect of the season on the TPC value of the AQ2 extracts (Kruskal–Wallis, $\chi^{2}$ = 9.30, *p*-value ≤ 0.01; Figure 3) showed that the AQ2 extracts from summer showed the highest TPC value, which was significantly higher than those observed for the AQ2 extracts from autumn and winter (Dunn test, *p*-value ≤ 0.05; Figure 3). The effect of the season on the TPC yield of the extracts AQ3 and AQ4 also showed statistical differences (Kruskal–Wallis, $\chi^{2}$ = 14.15, *p*-value ≤ 0.01; Figure 3). In particular, the TPC yields for both the extracts were higher in the summer samples, and they were significantly higher than the values obtained for these extracts from the spring and autumn samples (Dunn test, *p*-value ≤ 0.05; Figure 3). There was also a statistical difference in the effect of the season on the TPC value of the EA extracts (Kruskal–Wallis, $\chi^{2}$ = 10.24, *p*-value ≤ 0.05; Figure 3). The EA extract from the summer was the one with the highest TPC yield, which was significantly higher than those obtained for the EA extracts from winter and spring (Dunn test, *p*-value ≤ 0.05; Figure 3).

Brown algae belonging to the *Fucus* genus have been shown to have a high phenolic content in previous studies. *F. vesiculosus* aqueous extract has exhibited relatively high amounts of polyphenols, having an extra amount of 0.19 g of phloroglucinol equivalent per 100 g of extract when compared to other brown algae, *Ascophyllum nodosum* [22]. Ethanolic extracts of *F. serratus* have also been shown to have a phenolic content of 75.96 ± 10.11 mg GAE/g extract, which is significantly higher than the phenolic contents displayed by the other algae that were studied; there was 1.39 ± 0.24 mg of GAE/g extract and 4.76 ± 0.17 mg of GAE/g extract present in *Laminaria digitata* and *Gracilaria gracilis*, respectively [23]. In a study regarding fractioned extracts of *F. spiralis*, the aqueous extract showed the lowest phenolic content (8.00 ± 1.10 PGE/g extract), while the methanolic and dichloromethane extracts had the higher phenolic contents (379.00 ± 34.0 and 419.00 ± 3.00 PGE/g extract, respectively) [24]. This coincides with the results that were obtained in this study, where the aqueous extracts, specifically AQ4 from the summer, autumn, and spring samples, and the ethanolic extract Et80 for the winter samples, have the least amount of polyphenols between all of the extracts from *F. spiralis*, while the extracts that were obtained with a polar aprotic solvent, in this case ethyl acetate instead of dichloromethane, exhibited a higher phenolic content that ranged between 49.17 and 308.63 mg GAE/g of extract.

Similarly to what was observed in the present study, Jégou et al. [25] observed a seasonal variation of phlorotannin levels in brown algae, where the phloroglucinol content from the *Sargassaceae* species was higher toward the autumn months. This may be due to the build-up of phenolic compounds during the reproductive period of the algae, as noticed by Stiger et al. [26], who reported maximum phenolic contents in the summer and minimum phenolic contents in the winter. *Fucus vesiculosus* has been shown to have the lowest phenolic content in the spring, when there is an enlargement of the conceptacles, followed by an increase in the phenolic compounds, corresponding to the pre-fructification period, and reaching a maximum in the late summer, during the sterility period [27].

This difference between the aqueous and ethyl acetate extracts may be due to the fact that the algae from the *Fucus* genus are some of the brown algae with the highest amount of phenols, usually in the form of phlorotannins, even reaching up to 15% of their dry weight [3,28]. When these phlorotannins are the target compound of the extraction, moderately polar solvents yield better results [24]. This was also observed by Babbar et al. [29], where the polarity of the solvent influenced the TPC of the extracts, revealing that ethyl acetate was better than chloroform and n-hexane for the extraction of phenol-enriched extracts. This was probably due to their higher polarity and better solubility for phenolic components in algae extracts. Li et al. [30] indicated that after liquid–liquid extraction, the phenolic content in the ethyl acetate extracts was higher than the content of the initial ethanolic extract, while the TPC in the aqueous extracts was lower, as also observed in this study.

#### 2.1.2. Proton Nuclear Magnetic Resonance (^1^H NMR) Spectroscopy Analysis

**Figure 4:**
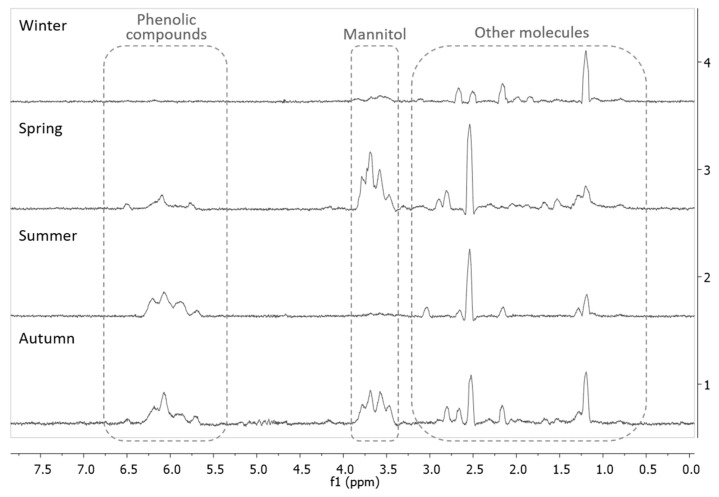
Stacked ^1^H NMR spectra of the EA extracts of the four seasons. Spectra acquired in D_2_O with water suppression.

The EA fractions, which presented the higher contents in phenolic compounds for all the seasons, were analyzed by NMR spectroscopy. The ^1^H NMR spectra of the EA extracts that were obtained for the four seasons, are presented in Figure 4. The peaks associated with aromatic compounds (such as phenolic compounds, including phlorotannins), found between 5.5 and 6.5 ppm, were more abundant in the summer EA extract, which is in agreement with the results that were obtained for the total phenolic content. Apart from the peaks associated with aromatic compounds, other peaks between 3.5 and 4 ppm could be observed, which were assigned to the presence of mannitol (peaks), and other compounds such as lipids (between 0.5 and 3 ppm) [21,31,32].

These results are similar to the ones found by Gager et al. [32], when analyzing the ^1^H NMR spectra of extracts from brown macroalgae, where *F. serratus* showed signals that corresponded to phenolic compounds (around 6 ppm), peaks that correspond to mannitol (4–3.5 ppm), and other peaks (below 3 ppm) that belong to other apolar molecules. Gall et al. [21] also found similar peaks that were assigned to aromatic compounds (5.5–6.5 ppm) and to mannitol (4–3.5 ppm), in brown algae from the Fucales order. Other species of brown algae also demonstrated different peak intensity and shape depending on the season; the profiles for *A. nodosum* and *H. siliquosa* had different peaks for the autumn and winter samples, which is a difference that can also be observed in the spectra presented by the *F. spiralis* samples of this study, due to the seasonality of the phenolic contents of these algae [33].

The quantification of phlorotannin content is not an easy task. The Folin–Ciocalteu method used for the analysis of TPC is the most common method used, but it is subject to interferences since the reagent used in the test also oxidizes several non-phenolic compounds, leading to over-estimations. This kind of method also detects the whole pool of phlorotannins present in the sample, without further characterization. The NMR method can detect very specific compounds, making it an innovative and rapid method to quantify phlorotannins in brown macroalgae, due to its specificity towards targeted molecules [25]. However, further purification steps of the phlorotannin-enriched extracts would be needed, in order to perform a more detailed structural characterization, which is not the aim of this study.

#### 2.1.3. Reversed-Phase High-Performance Liquid Chromatography (RP-HPLC) Analysis

Algal extracts contain a large number of very similar compounds that make the identification of individual structures very difficult, and although HPLC cannot be used to fully identify the structure of the phlorotannin and the position of the linkages, valuable information on the distribution, type, and size of phlorotannins can be obtained [33].

The application of liquid chromatography allows the large-scale isolation and purification of individual compounds. However, the literature concerning suitable HPLC methods, designed to identify and isolate brown algal phlorotannins, is scarce. A better separation is beneficial, since it means more purified individual compounds, resulting in better ionization and identification [34]. Once a compound of previously known beneficial activities is properly identified and isolated, it not only can be applied to food products as a functional ingredient, but it may also make the extraction of specific phlorotannins, with antioxidant abilities on an industrial scale, a reality.

**Figure 5:**
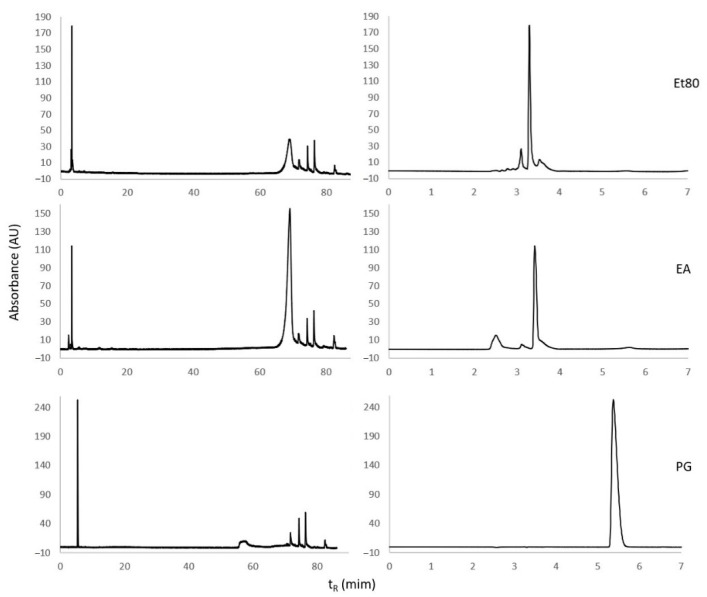
Chromatograms of Et80 and EA extracts and of phloroglucinol (PG), using RP-HPLC with a C18 column and UV detection at 280 nm. The chromatograms on the right are amplifications of the chromatograms of the left, in the range t_R_ = 0 to 7 min.

A tentative identification of phlorotannin compounds in the Et80 and EA extracts from the summer season was carried out using RP-HPLC, following the conditions that were described in Olate-Gallegos et al. [35], for the identification of polyphenols from brown macroalgae extracts. The UV chromatograms of the Et80 and EA extracts from the summer season are presented in Figure 5. The Et80 extract at 280 nm presents two peaks, with retention times between 3 and 4 min, which were assigned to low-molecular-weight phenolic compounds [35]. A broad peak at ca. 69 min was tentatively assigned to high-molecular-weight phlorotannins. The EA extract presents the same composition as the Et80 extract, but the peaks assigned to low-molecular-weight compounds are less intense, while the peaks assigned to high-molecular-weight compounds appear more intense. This is consistent with the EA extract being enriched in simpler phlorotannins, comparatively to the Et80 extract. A pure sample of phloroglucinol was injected in order to determine the respective retention time, which was 5.4 min. The algal extracts did not exhibit the monomer phloroglucinol. The peaks beyond 70 min were attributed to unknown low-polarity impurities from the algal extracts, which contaminated the injector system of the HPLC equipment.

The above results are consistent with what is reported in the literature. For instance, Steevensz et al. [36] reported that *F. vesiculosus* has displayed high concentrations of low-molecular-weight phlorotannins (<1200 Da). Melanson and Mackinnon [37] also found peaks of high intensity with a retention time inferior to 10 min, with these peaks being attributed to phlorotannins.

The difficulty of separating and identifying phlorotannins by HPLC stems from the conjunction of the following three factors: (i) isomeric phlorotannins may present identical retention times; (ii) phlorotannins of very low molecular weight are typically very polar compounds, and tend to exhibit short retention times under reversed-phase conditions, while intermediate and high-molecular-weight phlorotannins may exhibit much longer retention times, making separation difficult [23,33]; and (iii) structurally speaking, phlorotannins are a very diverse class of compounds. While Olate-Gallegos et al. [35] managed to separate and tentatively identify a myriad of phlorotannins that were extracted from different alga by reversed-phase HPLC-MS, the confirmation of the identity of a given phlorotannin compound still requires the use of pure analytical references. Due to the factors mentioned above, the availability of analytical references of phlorotannins is scarce.

### 2.2. Antioxidant Capacity of Extracts

#### 2.2.1. Ferric Reducing Antioxidant Power (FRAP)

Due to the presence of different bioactive compounds with anti-oxidative potential in the extracts, different methods have been used to investigate antioxidant activity in recent years. In this work, the antioxidant activity and polyphenol content were assessed by FRAP and DPPH.

**Figure 6:**
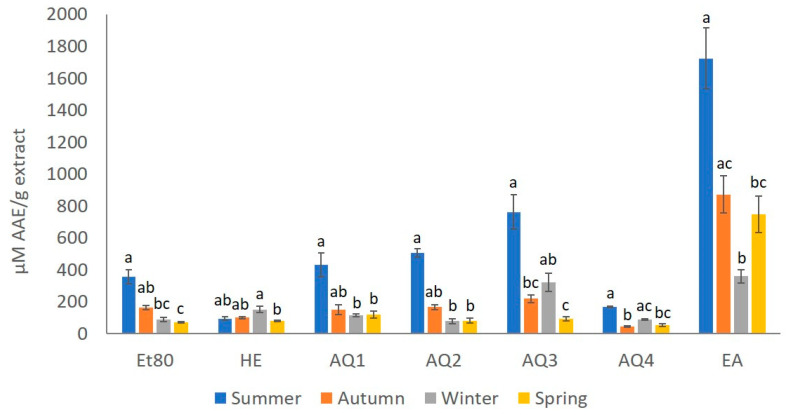
Ferric reducing antioxidant power (µM EAA/g extract) of *F. spiralis* extracts for all the seasons. Results are represented as mean ± SD (*n* = 4) and different lowercase letters represent significant differences (Dunn test, *p*-value ≤ 0.05) between seasons for each extract.

The ferric reducing antioxidant power (FRAP) assay is based on the ability of phenolic compounds to reduce Fe^3+^ to Fe^2+^. The redox reaction occurs in the presence of 2,4,6-trypyridyl-s-triazine, to induce the formation of a colored Fe^2+^ complex, allowing the colorimetric quantification of the of the phenolic compounds present in the sample, in comparison with a standard of known concentration. The analysis of the antioxidant activity of the extracts using the FRAP assay is presented in Figure 6.

The extract that gave the better results, considering the FRAP test for every season, was the EA extract, with 1723.00, 873.38, 360.25, and 748.38 µM EAA/g of extract for the summer, autumn, winter and spring seasons, respectively. The extracts with the lower FRAP values were He from summer, with 96.25 µM EAA/g of extract; AQ2 from winter, with 79.19 µM EAA/g of extract; and AQ4 from autumn and spring, with 45.59 and 54.90 µM EAA/g of extract, respectively.

The season with the best results in the FRAP assay, for every extract except He, was summer, and the season with the lowest values was spring, since most of the extracts from this season, Et80, He, AQ2, AQ3 and AQ4, presented values of less than 100 µM EAA/g of extract (Figure 6).

The results that were obtained for the effect of the seasons on the FRAP values of the extracts showed statistically significant differences (Kruskal–Wallis, $\chi^{2}$ = 54.17, *p*-value ≤ 0.01; Figure 6). Thus, it was possible to observe that the FRAP of the extracts from the summer season was significantly different from most of the seasons (Dunn test, *p*-value ≤ 0.05; Figure 6). When analyzing the effect of the seasons on the FRAP values of the Et80 extracts, it was possible to observe statistical differences (Kruskal–Wallis, $\chi^{2}$ = 13.83, *p*-value ≤ 0.01; Figure 6). The extracts from the summer season presented the higher FRAP values, which were significantly higher than those that were obtained for spring extracts, with the lower FRAP values (Dunn test, *p*-value ≤ 0.05; Figure 6). The season also had a statistically significant effect on the FRAP values of the He extracts (Kruskal–Wallis, $\chi^{2}$ = 11.73, *p*-value ≤ 0.01; Figure 6). Unlike what was observed for the other extracts, He from summer did not show the highest FRAP values, but instead, the winter He extract presented a FRAP value that was significantly higher than the lowest FRAP value that was obtained for the spring He extract season (Dunn test, *p*-value ≤ 0.05; Figure 6). The effect of the season on the FRAP values for the extracts AQ1 and AQ2 showed statistical differences (Kruskal–Wallis, $\chi^{2}$ = 12.75, *p*-value ≤ 0.01; Figure 6). The AQ1 and AQ2 extracts from summer presented FRAP values that were significantly higher than those that were obtained for the same extracts from winter and spring (Dunn test, *p*-value ≤ 0.05; Figure 6). For the AQ3 and AQ4 extracts, the season also showed a statistically significant effect on the FRAP values of the extracts (Kruskal–Wallis, $\chi^{2}$ = 13.52, *p*-value ≤ 0.01; Figure 6). Higher FRAP values for these extracts were obtained for the summer samples, and these FRAP values were significantly higher than those that were obtained for the same extracts from spring and autumn (Dunn test, *p*-value ≤ 0.05; Figure 6). The effect of the season on the FRAP values of the EA extract showed statistical differences (Kruskal–Wallis, $\chi^{2}$ = 13.06, *p*-value ≤ 0.01; Figure 6). The EA extract from summer season was also the one with the highest FRAP value, which was significantly higher than those obtained for the EA extracts from winter and spring (Dunn test, *p*-value ≤ 0.05; Figure 6).

#### 2.2.2. 1,1-Diphenyl-2-picryl-hydrazyl (DPPH) Free Radical Assay

The DPPH (1,1-Diphenyl-2-picryl-hydrazyl) free radical assay is based on the scavenging capacity of antioxidants towards the reduction in the colored DPPH free radical. The reduction in color is proportional to the percentage reduction in the radical, by the antioxidant molecules that are present in the sample.

**Figure 7:**
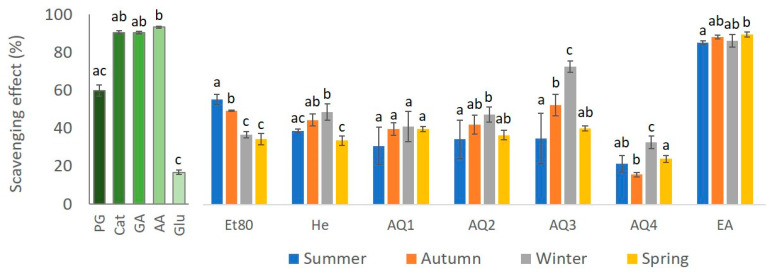
DPPH scavenge ability of *F. spiralis* extracts from the four seasons, compared to standards phloroglucinol (PG), catechol (Cat), gallic acid (GA), ascorbic acid (AA) and glucose (Glu). Results are represented as mean ± SD (*n* = 4) and different lowercase letters represent significant differences (Dunn test, *p*-value ≤ 0.05) between seasons for each extract, and between standards.

The antioxidant activity of several standards was determined using the DPPH method, in order to compare with the results obtained for the extracts. The results are presented in Figure 7. The standards had a high percentage of reduction in DPPH, with ascorbic acid (AA) being the best with a reductive capability of 93.2%, followed by catechol (Cat), gallic acid (GA), and phloroglucinol (PG), with reductive percentages of 90.43%, 90.35%, and 59.9%, respectively. The standard with the lowest reductive capability was glucose (Glu), with a 16.8% reduction in DPPH. For all the seasons, the EA extract was the one that showed the highest DPPH reductive capability, with a DPPH reduction percentage of 85.1% for the EA from the summer samples, 88.1% for the autumn EA extract, 86.1% for the winter EA extract, and 89.4% for the spring EA extract. Thus, the EA fractions of all the seasons showed a DPPH reductive capability that was almost as high as the three best standards. On the other hand, extract AQ4 showed the lowest percentages of DPPH reduction for all the seasons, with 21.6% for AQ4 from summer, 15.7% for AQ4 from autumn, 32.6% for AQ4 from winter, and 23.97% for AQ4 from spring. However, with the exception of the autumn AQ4 extract, the percentages shown by this extract in the remaining seasons are still higher than the percentage reduction in DPPH displayed by the standard glucose (16.8%).

The results that were obtained for the effect of the seasons on the reduction percentage of the DPPH compound showed statistically significant differences (Kruskal–Wallis, $\chi^{2}$ = 8.08, *p*-value ≤ 0.05; Figure 7). The EA extract had a higher scavenging effect of the DPPH radical for every season. These differences were statistically significant for most of the comparisons between the extracts (Dunn test, *p*-value ≤ 0.05; Figure 7).

The effect of the seasons on the reduction percentage of the DPPH compound by the Et80 extracts showed statistical differences (one-way ANOVA, F-value = 86.79, *p*-value ≤ 0.01; Figure 7). The season with the higher percentage of reduction in the compound was summer, followed by autumn, and both the seasons had a significantly higher scavenging ability than winter and spring (Tukey test, *p*-value ≤ 0.01; Figure 7).

A statistical difference could be seen in the analysis of the effect of the seasons on the DPPH scavenging effect of the He extract (one-way ANOVA, F-value = 20.54, *p*-value ≤ 0.01; Figure 7). The season with the highest DPPH reductive ability for this extract was winter, followed by autumn. Also, all these seasons had a significantly higher scavenging effect than spring (Tukey test, *p*-value ≤ 0.01; Figure 7).

Regarding the effect of seasons on the scavenging effect of the DPPH radical of the AQ1 extracts, no statistical differences were observed (one-way ANOVA, F-value= 2.12, *p*-value > 0.05; Figure 7).

The effect of seasons on the scavenging effect of the DPPH radical of the AQ2 extracts showed statistical differences (one-way ANOVA, F-value= 3.70, *p*-value ≤ 0.05; Figure 7). The season with the highest reduction percentage for this extract was winter. This season had a significantly higher scavenging ability than the other seasons (Tukey test, *p*-value ≤ 0.05; Figure 7).

When analyzing the effects of the seasons on the scavenging effect of the AQ3 extracts on the DPPH compound, a statistical difference could be seen (one-way ANOVA, F-value = 20.51, *p*-value ≤ 0.01; Figure 7). The season with the highest reduction percentage for this extract is winter, followed by autumn. These seasons had a significantly higher scavenging activity than summer and spring (Tukey test, *p*-value ≤ 0.05; Figure 7).

The effect of the season on the scavenging effect of the AQ4 extracts on the DPPH compound also presented significant differences (One-way ANOVA, F-value = 22.86, *p*-value ≤ 0.01; Figure 7). The season with the highest scavenging activity for this extract was winter, followed by spring. These seasons had a significantly higher reduction percentage than summer and autumn (Tukey test, *p*-value ≤ 0.01; Figure 7).

The effect of the season on the DPPH radical scavenging effect of the EA extracts showed statistical differences (Kruskal–Wallis, $\chi^{2}$ = 10.10, *p*-value ≤ 0.05; Figure 7). The season with the highest reduction percentage of the radical for this extract was spring. This season had a significantly higher scavenging ability than the other seasons (Dunn test, *p*-value ≤ 0.05; Figure 7).

**Figure 8:**
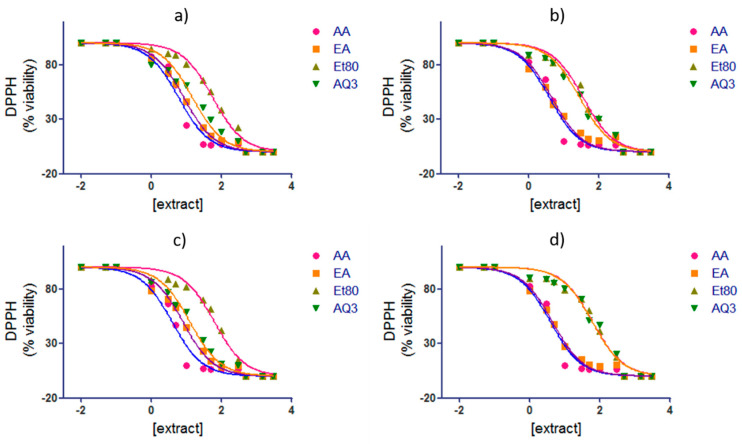
IC_50_ of *F. spiralis* extracts Et80, EA and AQ3, from (**a**) summer; (**b**) autumn; (**c**) winter and (**d**) spring, in comparison to ascorbic acid (AA) standard.

The concentration of the extracts, necessary to reduce 50% of the DPPH compound, was determined for the extracts of the four seasons that presented a higher radical scavenging ability (Et80, EA, and AQ3). The IC_50_ for ascorbic acid was also performed for comparison. The results are shown in Table 1 and Figure 8.

For all of the seasons, the extract with the lowest concentration necessary to reduce 50% of DPPH was the EA extract, with a concentration of 8.41 µg/mL for the summer season, 4.49 µg/mL for the autumn season, 7.87 µg/mL for the winter season, and 4.45 µg/mL for the spring season. The extract with the highest concentration needed to reduce half of the DPPH compound was Et80, with the following concentrations: 56.19, 35.24, 61.73, 61.40 µg/mL for the summer, autumn, winter, and spring season, respectively.

In a study by Wang et al. [38], when analyzing the IC_50_ of the ethyl acetate and n-butanol extracts of *F. vesiculosus* and standards, the EA extract had a lower IC_50_ concentration than the latter; they were 3.76 ± 0.22 and 4.77 ± 0.25 µg/mL, respectively. However, the ascorbic acid and BHT standards had a lower IC_50_, of 2.49 ± 0.06 and 3.28 ± 0.09 µg/mL, respectively [38]. These results are consistent with those of the present study, where the EA extracts had a relatively low IC_50_ when compared to the other extracts of *F. spiralis* that were analyzed, and the standard ascorbic acid had an even lower concentration necessary to scavenge 50% of the DPPH in the samples.

### 2.3. Relation between Phenolic Compounds Content and Antioxidant Capacity

Previous studies have examined the relation between the antioxidant activity of an algae extract and its phenolic content. Heffernan et al. [23] found that macroalgae extracts from Atlantic coasts that have previously shown a high polyphenol content, also showed strong ferric reducing antioxidant power. Ethanolic extracts of *F. serratus* exhibited a TPC of 75.96 ± 10.12 µg of GAE, while ethanolic extracts of *Laminaria* sp., *Codium* sp., and *Gracillaria* sp. only contained 1.39 ± 0.24, 2.40 ± 0.50, and 4.76 ± 0.17 µg of GAE, respectively. When the ferric reducing power of these extracts was analyzed, *F. serratus* showed 78.30 ± 15.53 µg of Trolox equivalents, while the other three extracts showed 8.51 ± 0.31, 6.01 ± 0.29, and 4.76 ± 0.16 µg TE, respectively. Agregán et al. [39] saw a positive correlation between the TPC and the antioxidant activity of extracts from macro- and microalgae, where the ethanolic extract of *F. vesiculosus,* which had a TPC of 20 g of phloroglucinol equivalent per 100 g of extract, showed a ferric reducing activity of 3.45–3.82 µmol of Trolox equivalent per gram of dry weight. In contrast, the ethanolic extract of *Chlorella* sp. contained only 4.5 g of PGE/100 g of extract, and exhibited a relatively low ferric reducing activity of 0.62 µmol TE/g DW. Similarly, the results obtained in this study, showed a relation between the content of polyphenols in the extracts from *F. spiralis* and their ferric reducing antioxidant power. The ethyl acetate extracts presented the highest phenolic content. Consequently, these also presented the highest antioxidant activity during FRAP testing. Conversely, the aqueous and hexane extracts showed not only a low polyphenolic content, but also a low reducing activity.

Other studies have reported a difference in the FRAP activity between different extracts of *F. spiralis*. Tierney et al. [40] noticed that if the solvent used for the extraction had yielded high results in TPC, the extract would also exhibit high ferric reducing activity. In that study, the ethanolic extract of *F. spiralis* displayed a lower amount of polyphenol content than the methanolic extract; they were 37.03 ± 3.01 and 39.04 ± 5.72 µg of phloroglucinol equivalent per mg of sample, respectively, and the ferric reducing power of the EtOH extract was also lower than the MeOH extract; they were 20.64 ± 2.19 and 25.63 ± 0.63 µg Trolox equivalent per mg of sample, correspondingly.

The correlation between TPC and FRAP seen in this study may be due to the rich content of phlorotannins with a high molecular weight of the ethyl acetate extracts [41,42], since ethyl acetate has been widely used to selectively extract polyphenolic compounds of intermediate polarity from various vegetal and algal samples [38]. These phlorotannins that are present in the algae from the *Fucus* genus have antioxidant activity that was previously associated with their molecular skeleton, since their phenol rings act as electron traps to scavenge peroxyl, superoxide anions and hydroxyl radicals [43], thus conferring a strong antioxidant ability, as seen in this study.

Comparably to the results obtained in the ferric reducing antioxidant power, the *F. spiralis* extracts with a high polyphenol content also showed the best scavenging activity. Wang et al. [38] reported that the ethyl acetate fractions from the ethanolic extract of *F. vesiculosus* exhibited the highest scavenging activity, as well as the highest levels of TPC, while the aqueous and n-hexane fractions were less effective and had the lowest phenolic content, as can be seen in this study. Hermund [12] reported that the ethanolic extract of *F. vesiculosus* had a lower polyphenol content than its ethyl acetate fraction, 20.4 ± 2.4 and 26.5 ± 1.2 g GAE/100 g DW, respectively, and that the DPPH radical scavenging capacity of the ethyl acetate fraction was also higher than that of the ethanolic fraction. A positive correlation between TPC and DPPH scavenging activity was reported, but it was not proportional, since the TPC of the ethyl acetate fraction was twice as high as the TPC of the water extract, but the scavenging activity was ten times higher. Similarly, the ethyl acetate fraction from the *F. spiralis* extract that was analyzed in this study had higher reducing activity of the DPPH compound for every season. This is in agreement with the fact that extracts with high radical scavenging capacity have also shown high reducing power [11].

Polyphenols are well known to show biological and antioxidant activity. Gallic acid is a naturally occurring phenol that is a strong antioxidant and more effective than other soluble antioxidants, and its antioxidant activity has often been compared to that of ascorbic acid [44]. Other antioxidants that are present in brown algae have demonstrated antioxidant abilities [45]. Structurally simple phenols that are present in brown algae have one hydroxyl group (-OH), catechol and benzenediols have two -OH groups, while phloroglucinol and benzenetriols have three hydroxyl groups. This polyphenolic content is associated with the antioxidant activity of the seaweed [46]. Apart from polyphenols, brown algae also contain sulphated polysaccharides (fucans) that are mainly composed of fucose and sulphate groups, but may also contain amounts of other sugars, such as glucose, in their composition. These molecules are of economic importance, and have also demonstrated their potential as free-radical scavengers and antioxidants, for the prevention of oxidative damage in living organisms [47].

In this study, the comparison of the scavenging activity of the extracts of *F. spiralis* was carried out against two strong antioxidant standards (ascorbic and gallic acid), two phenols of increasing complexity (catechol and phloroglucinol), and against a polysaccharide (glucose). Ascorbic and gallic acids and catechol had the best results, having more than a 90% reduction in the DPPH compound. Phloroglucinol had a lower reducing activity compared to ascorbic acid, gallic acid and catechol, having no more than a 60% reduction in DPPH; while glucose showed the lowest reduction percentage of the standards (lower than 20%). It is indicated that the effectiveness and bioactivity of the polyphenols depends on the resonance stabilization of the phenoxy radical, which is influenced by the number of substituents (relative to the hydroxyl group) attached to the aromatic ring at the *ortho* and *para* positions. As stated before, phlorotannins have complex polymeric structures, with up to eight interconnected rings that are derived from phloroglucinol monomer units, making them the most potent free radical scavenger from polyphenols [46]. This explains why the EA extracts from *F. spiralis*, which are phlorotannin-enriched, have the highest percentages of reduction in the DPPH compound, for all of the seasons, while the aqueous and n-hexane extracts had lower percentages, but no lower than that of glucose. This may be due to the presence of other compounds, such as pigments, proteins or peptides, which may interfere with the scavenging ability of the extracts [11], and to the low content of phlorotannins in them. This is in agreement with the results that were obtained by Li et al. [30], who analyzed the DPPH radical scavenging activity of crude ethanol extracts and its fractions of brown algae, where the IC_50_ of the ethanolic extract was 150.13 µg/mL and the IC_50_ of the ethyl acetate fraction, which was the lowest, was 14.61 µg/mL; while the aqueous extract had the higher IC_50_ value analyzed, which was 206.15 µg/mL. Wang et al. [38] evaluated the DPPH radical scavenging activity of different solvent fractions of *F. vesiculosus*, and compared them with standard antioxidants BHT, α-tocopherol, and ascorbic acid. Ethyl acetate extracts had the higher inhibition percentage in comparison with the hexane, aqueous and n-butanol extracts, similarly to the results presented in our study, where the EA extracts of every season certainly have a higher reduction percentage than the n-hexane and aqueous extracts of *F. spiralis.*

In general, algae are exposed to extreme environmental conditions, dangerous UV radiation, low nutrient availability, salinity, and temperature, all of which will induce the formation of oxidizing agents that act as free radicals and other reactive species that can potentially interact with biological systems. As such, algae may present resistance to serious structural and photodynamic damage during metabolism. This resistance may be due to the production of various metabolites, including phenolic compounds, which are known as effective reducing agents and free radical scavengers [18]. The biochemical composition of marine algae is known to be highly influenced by geographical location, environment, season, and sampling conditions. Fellah et al. [48] found that there was a seasonal variation in phenolic compounds content, as well as in the antioxidant activity of the two species of brown algae that were analyzed, similar to the one presented in this study, where the phenolic content of the *F. spiralis* extracts was higher for the summer season. This seasonal fluctuation has also been found in other species of the *Fucus* genus. Mendes et al. [49] found a monthly variation in the phenolic content of *F. vesiculosus*, having the lowest values of TPC for the winter months and the highest during the spring, summer, and autumn. Paiva et al. [1] also found seasonal variation in the methanolic and acetone:water extracts of *F. spiralis*, not only in the protein, carbohydrate and lipidic composition of the samples, but also in the TPC of the extracts for the summer and winter seasons, obtaining a lower phenolic content in the acetone:water extracts during the winter season, but a lower phenolic content in the methanolic extract for the summer season. However, the difference present in the phenolic content between both seasons suggests that the production of phlorotannins, by members of the genus *Fucus*, is correlated with UV radiation [1], which is in accordance with the results that were observed in this study, where the *F. spiralis* extracts that were obtained from samples collected during the summer had higher values of TPC. This increase in phenolic compound during the summer months has been associated with a photoprotective mechanism with dynamic photoinhibition of photosynthesis, in order to tolerate light stress in response to the intensified UV radiation [49].

Since *F. spiralis* is an intertidal species, it is consequently often exposed to high levels of solar radiation in the summer, leading to a development of a physiological adaptation, similarly to the synthesis of a UV protector, to tolerate this condition of increased radiation and photoperiods. Mancuso et al. [50] also saw a relation between increasing seawater and air temperatures, and the synthesis of the phenolic compounds of other intertidal brown algae, finding that the phenolic content of algal extracts also increases when the temperature increases above 25 °C, with the higher values found at 28 °C. This hypothesized that the phenolic content present in this intertidal alga is affected by the thermal seawater conditions that are experienced during summer, and that there are physiological responses to ambient temperature variation. Grazing pressure also seems to affect phenolic content, since their presence deters herbivores [50]. There is a higher incidence of grazing during the summer and early autumn months, when there is less growth and more carbon available for defense chemicals [49].

There also seems to be a relation between phenolic content and the reproductive cycle of the algae. *F. vesiculosus* showed higher contents of phenols during the fertile periods of the summer and autumn; while showing a lower TPC for the winter [51]. Vandanjon et al. [52] also found a biochemical variation in brown algae depending on its life cycle, finding a higher percentage of TPC during the months of June and July, during their reproduction stage. In the present study, *F. spiralis* also showed a higher polyphenolic content during the summer months, which is the reproductive season of this algae [53].

## 3. Materials and Methods

### 3.1. F. spiralis Collection and Preparation

*F. spiralis* was collected in the four seasons (autumn, winter, spring and summer) during the years of 2018 and 2019, in the upper intertidal zone of the Marques Neves Beach (39°22′13.5′′ N 9°23′14.8′′ W), Peniche (Portugal), during low tide. The algae were washed with salt water (35% (*w*/*v*)), frozen at −80 °C and freeze-dried (Labogene, CoolSafe 55-4). The biomass was then finely crushed with a laboratory blender (Bimby vorwerk, thermomix 31-1) and stored in a dry recipient in the absence of light until further studies.

### 3.2. Chemicals

Extra-pure grade solvents used for the extractions were purchased from commercial suppliers. Folin–Ciocalteu reagent, gallic acid, DPPH (2,2-diphenyl-1-picrylhydrazyl), TPTZ (2,4,6-Tri (2-pyridyl)-s-triazine), phloroglucinol, pyrocatechol, ascorbic acid, and glucose were obtained from Sigma-Aldrich (St. Louis, MO, USA).

### 3.3. Extraction Procedure

The extraction procedure described below was performed for *F. spiralis* biomass obtained in the four seasons.

#### 3.3.1. Crude Ethanolic Extracts

Firstly, 30 g of biomass were suspended in 240 mL of an EtOH/H_2_O mixture (80:20 *v*/*v*) (biomass:solvent ratio 1:10 *w*/*v*). The suspension was mixed thoroughly for 30 min with the help of a magnetic stirrer, at room temperature. After extraction, the suspension was vacuum filtered with a qualitative paper filter (filter paper VWR 2–3 µm). The solvent was removed by reduced pressure using a rotary evaporator. The dried extract (Et80) was weighted and stored until further tests.

#### 3.3.2. Liquid–Liquid Semi-Purification of the Crude Ethanolic Extracts

Crude ethanol–water (80:20) extracts (Et80) of *F. spiralis* were fractionated using a liquid–liquid separation procedure based on polarity adapted from Gall et al. [21], in order to obtain phlorotannin-enriched fractions. In a centrifuge tube, 1.5 g of the Et80 extract was dissolved in 40 mL of distilled water. Then, 40 mL of n-hexane was added and the mixture was shaken in a separating funnel, obtaining two phases, an aqueous phase (AQ1) and an n-hexane phase (He). The aqueous phase AQ1 was extracted using the same amount of n-hexane two more times. The n-hexane phases (He) were combined and the solvent was removed by reduced pressure using a rotary evaporator. The aqueous extract AQ1 was reduced to about 50 mL using a rotary evaporator. A sample of 10 mL of AQ1 was taken, the solvent was removed under reduced pressure and the residue was stored for analysis. To the remaining 40 mL of AQ1, 80 mL of ethanol was added and the mixture was thoroughly mixed for 15 min and stored at −20 °C for 24 h. The mixture was then centrifuged at 8000 rpm and 4–5 °C for 20 min. The pellet was discarded, and the process was repeated until no pellet was obtained after centrifugation. Ethanol was completely evaporated to give the second aqueous phase (AQ2). A sample of 10 mL of AQ2 was taken, the solvent evaporated and the residue kept for further analysis. A volume of ca. 120 mL of acetone was added to 40 mL of the aqueous phase AQ2, the mixture was thoroughly mixed for 15 min and stored at −20 °C for 24 h. The mixture was centrifuged at 8000 rpm and 4–5 °C for 20 min. The pellet was again discarded, and the process was repeated until no pellet was obtained after centrifugation. Once there was no pellet, the acetone was removed using a rotary evaporator, and the third aqueous phase (AQ3) was obtained. Then, 10 mL of aqueous phase AQ3 was taken, the solvent was evaporated, and the residue stored for further analysis. Further, 40 mL of ethyl acetate was added to the aqueous phase AQ3 resulting in two phases, an aqueous phase (AQ4) and an ethyl acetate phase (EA). The aqueous phase was extracted two more times using the same volume of ethyl acetate. The EA phases were combined, and the EA and AQ4 phases were evaporated to dryness with the help of a rotary evaporator.

### 3.4. Characterization of the Extracts

#### 3.4.1. Total Phenolic Content (TPC)

The content of phenols in the sample was determined using the method described by Li et al. [30] with some modifications. The determination of TPC was done using a 96-wells plate, putting 2 µL of the sample, 158 µL of distilled water and 10 µL of Folin reactive in each well. After complete dissolution, it was left to incubate for 2 min at room temperature. Then, 30 µL of Na_2_CO_3_ 20% was added to each well and the mixture was left to incubate for one hour at room temperature, after which the absorbance was read at 755 nm. Four replicates were conducted for each extract/standard concentration. Samples of the extracts with a concentration of 5 mg/mL (EtOH/H_2_O, 80:20 *v*/*v*) were used. Controls were prepared by replacing the sample with distilled water. A standard curve using gallic acid was created, using solutions with the following concentrations: 1, 0.3, 0.1, 0.03, and 0.01 mg/mL. The TPC is expressed as mg of gallic acid equivalents per gram of extract (GAE/g extract).

#### 3.4.2. Proton Nuclear Magnetic Resonance (^1^H NMR) Spectroscopy

^1^H NMR spectra of the ethyl acetate extracts were measured on a Bruker (Billerica, MA, USA) Avance III 400 MHz instrument equipped with a 5 mm broad-band diffusion probe (DifBB), using D_2_O as solvent, at a temperature of 298 K. The 1D ^1^H experiments were acquired with 32 transients with 65 K data points, in a spectral window of 8015 Hz centered at 1881 Hz, using the standard Bruker pulse sequence “zgesp” for water suppression. The chemical shifts were expressed in ppm, using the residual solvent peak as reference (in the standard proton experiment).

#### 3.4.3. Reversed-Phase High-Performance Liquid Chromatography (RP-HPLC)

The extracts were analyzed by reversed-phase HPLC (RP-HPLC), using a Merck-Hitachi Elite LaChrom HPLC (Hitachi High-Technologies Corporation, Tokyo, Japan) system equipped with an L-2450 DAD detector, an L-2200 autosampler and an L-2130 pump. Acquisition of the chromatograms was made with EZChrom Elite software v3.3.2 (Agilent Technologies, Santa Clara, CA, USA). The analyses were carried out at room temperature with an ACE Advanced Chromatography Technologies Ltd. HPLC C18 column (250 × 4.6 mm diameter size, 5 μm particle size, 100 Å pore size) equipped with a matching guard cartridge. The volume of injection was 70 µL. A gradient mobile phase consisting of formic acid in water (A, 1% *v*/*v*) and acetonitrile (B) was used, at a flow rate of 1 mL/min. The composition for a 90 min. run was as follows [35]: 0–5 min. 5% B; 5–60 min, 5–30% B (linear); 60–70 min B, 30–60% B (linear); 70–80 min, 60% B; 80–90 min, 60–5% B (linear). The detection of aromatic compounds was made at 254 and 280 nm.

### 3.5. Evaluation of the Antioxidant Capacity of the Extracts

#### 3.5.1. Ferric Reducing Antioxidant Power (FRAP)

The method used was described by Puspita et al. [8], with some variations. The following solutions were prepared: a 300 mM acetate buffer (A) (pH 3.6), TPTZ 10 mM (B), and a solution of FeCl_3_:6H_2_O (C). For the reagent TPTZ, the solutions A, B and C were mixed in a ratio of 10:1:1, and the mixture was kept away from light. The assay was done in a 96-well plate, mixing 5 µL of the sample and 195 µL of TPTZ reagent in each well. Four replicates were conducted for each extract/standard concentration. The plate was left to incubate at room temperature for 4 h and the absorbance was read at 593 nm. Samples of the extracts dissolved in EtOH/H_2_O (80:20 *v*/*v*) with concentrations of 1 mg/mL (Et80, AQ1, AQ2, and AQ3), 5 mg/mL (AQ4 and He) and 0.5 mg/mL (EA) were used. Controls were prepared by replacing the sample with distilled water. A standard curve using ascorbic acid was created using solutions with the following concentrations: 1000, 750, 500, 200 and 20 µM. The FRAP is expressed as µM of ascorbic acid equivalents per gram of extract (µM AAE/g extract).

#### 3.5.2. DPPH (2,2-Diphenyl-β-picrylhydrazyl) Radical Scavenging Activity

The DPPH radical scavenging activity was analyzed with the method according to Cruces et al. [54] with some modifications. DPPH solution was made dissolving 1.6 mg of DPPH in 40 mL of methanol. The assay was conducted using 96-well plates, with a control that consisted of eight replicates of 2 µL of ethanol and 198 µL of DPPH solution. Then, 2 µL of the sample and 198 µL of DPPH solution were mixed in each well, ensuring the homogenization of the solution. Four replicates were conducted for each extract. The plate was left to incubate at room temperature for 30 min, protecting it from light, and after that period the absorbance was read at 517 nm. Samples of the extracts with a concentration of 5 mg/mL (EtOH/H_2_O, 80:20 *v*/*v*) were used. Negative controls were prepared by replacing the sample with distilled water. The DPPH radical scavenging activity is expressed as the scavenging effect (reduction %) of the DPPH radical. Phloroglucinol, pyrocatechol, gallic acid, ascorbic acid, and glucose were used as reference standards (1 mg/mL). The antioxidant activity of the extracts was defined as the capacity to eliminate free radicals, and determined using Equation (1), as follows:

Scavenging effect (%) = [1 − (A_sample_-A_sample_blank_)/A_control_)] × 100 (1)

where A_control_ is the absorbance of the control (DPPH solution with ethanol), A_sample_ is the absorbance of the test sample (DPPH solution plus test sample), and A_sample_blank_ is the absorbance of the sample in methanol (sample without DPPH solution). IC_50_ was determined for the extracts presenting higher capacity to eliminate free radicals, Et80, EA and AQ3, using ascorbic acid as a comparative standard. The IC50 curve was obtained by determination of the DPPH radical scavenging activity of the selected extracts (EtOH/H_2_O, 80:20 *v*/*v*) with the following concentrations: 0.1; 0.3; 0.5; 1; 3; 5; 10; and 30 mg/mL. The results are presented as IC_50_ values (μg/mL).

### 3.6. Statistical Analysis

All data were checked for normality and homoscedasticity. Since they were not met, the non-parametric Kruskal–Wallis test was used, followed by Dunn multiple comparison test [55]. Whenever applicable, Dunnett’s multiple comparisons test was employed to determine significant differences relative to control treatment. To assess the differences between extracts and between seasons regarding the results obtained for TPC, FRAP and DPPH, a one-way analysis of variance (Kruskal–Wallis) was performed [55]. For the DPPH reductive test, the standards were compared with the extracts for each season and the same procedure was performed.

The half maximal inhibitory concentration (IC_50_) was calculated from nonlinear regression analysis using the software GraphPad InStat v5.1 (San Diego, CA, USA) with Equation (2) [56], as follows:

(2) $$y=100/\left(1+10^{\left[x-\log\left(IC_{50}\right)\right]}\right)$$

where ***y*** is the % of viability (% viability = 100 − % reduction), ***x*** is log[extract], and ***IC*_50_** is the concentration of extract needed to reduce half the DPPH radical.

The statistical analysis of the data was carried out using the software Rstudio AGLP v3. All the results were considered statistically significant at the 5% significance level (*p*-value ≤ 0.05) [57]. Results are presented as means ± standard deviation (SD).

## 4. Conclusions

The present study revealed a variability in the phenolic content, as well as the antioxidant properties, relative to the different extracts obtained from the extraction and subsequent fractionation of *F. spiralis*.

The EA extracts from this alga showed a higher total polyphenolic content, and, as expected, the iron chelating activity (FRAP) and DPPH radical scavenging activity were significantly higher in these extracts when compared to the others, showing that the extraction of enriched algal extracts using adequate solvents can increase the antioxidant activity of the samples.

Since biotic and abiotic factors may affect the production and concentration of antioxidant compounds in brown algae, seasonal variations can also be observed in the content of phenolic compounds, as well as the antioxidant activities of *F. spiralis* extracts. The extracts that were obtained from *F. spiralis* collected during the summer season showed a higher total phenolic content out of every season, and demonstrated the best iron chelating and DPPH scavenging activities as well.

The biochemical composition observed, as well as the high functional bioactivity values shown by the summer EA extract, provided information on the best extract and period of the year to collect this seaweed for its potential use as a natural antioxidant for food products.

## Figures and Tables

**Table 1:** IC_50_ of *F. spiralis* ET80, EA and AQ3 extracts from all the seasons, in comparison to the IC_50_ of ascorbic acid (AA) standard.

	IC_50_ (µg/mL)			
Extract	Summer	Autumn	Winter	Spring
AA	5.999 (5.195–6.928)	3.881 (3.373–4.465)	3.881 (3.373–4.465)	3.881 (3.373–4.465)
Et80	56.19 (51.11–61.78)	35.24 (31.62–39.28)	61.73 (54.39–70.07)	61.40 (54.69–68.93)
EA	8.414 (7.964–8.890)	4.486 (3.993–5.040)	7.872 (7.215–8.588)	4.450 (4.075–4.860)
AQ3	14.86 (12.93–17.08)	27.92 (25.14–31.01)	12.45 (11.40–13.59)	59.87 (51.94–69.01)

## Data Availability

Not applicable.

## Sample Availability

Sample of the compounds are not available from authors.

## References

[1] Paiva L., Lima E., Neto A.I., Baptista J. (2018). Seasonal variability of the biochemical composition and antioxidant properties of fucus spiralis at two Azorean Islands. Mar. Drugs.

[2] Kornprobst J.-M. (2014). Phaeophyceae (Brown Algae). Encyclopedia of Marine Natural Products.

[3] Imbs T.I., Zvyagintseva T.N. (2018). Phlorotannins are Polyphenolic Metabolites of Brown Algae. Russ. J. Mar. Biol..

[4] Kuda T., Nishizawa M., Toshima D., Matsushima K., Yoshida S., Takahashi H., Kimura B., Yamagishi T. (2021). Antioxidant and anti-norovirus properties of aqueous acetic acid macromolecular extracts of edible brown macroalgae. LWT.

[5] Javed A., Hussain M.B., Tahir A., Waheed M., Anwar A., Shariati M.A., Plygun S., Laishevtcev A., Pasalar M. (2021). Pharmacological Applications of Phlorotannins: A Comprehensive Review. Curr. Drug Discov. Technol..

[6] Honold P.J., Jacobsen C., Jónsdóttir R., Kristinsson H.G., Hermund D.B. (2016). Potential seaweed-based food ingredients to inhibit lipid oxidation in fish-oil-enriched mayonnaise. Eur. Food Res. Technol..

[7] Machu L., Misurcova L., Ambrozova J.V., Orsavova J., Mlcek J., Sochor J., Jurikova T. (2015). Phenolic content and antioxidant capacity in algal food products. Molecules.

[8] Puspita M., Déniel M., Widowati I., Radjasa O.K., Douzenel P., Marty C., Vandanjon L., Bedoux G., Bourgougnon N. (2017). Total phenolic content and biological activities of enzymatic extracts from *Sargassum muticum* (Yendo) Fensholt. J. Appl. Phycol..

[9] Karadağ A., Hermund D.B., Jensen L.H.S., Andersen U., Jónsdóttir R., Kristinsson H.G., Alasalvar C., Jacobsen C. (2017). Oxidative stability and microstructure of 5% fish-oil-enriched granola bars added natural antioxidants derived from brown alga *Fucus vesiculosus*. Eur. J. Lipid Sci. Technol..

[10] Miranda J.M., Trigo M., Barros-Velázquez J., Aubourg S.P. (2016). Effect of an icing medium containing the alga *Fucus spiralis* on the microbiological activity and lipid oxidation in chilled megrim (*Lepidorhombus whiffiagonis*). Food Control.

[11] Farvin K.H.S., Jacobsen C. (2015). Antioxidant activity of seaweed extracts: In Vitro assays, evaluation in 5% fish oil-in-water emulsions and characterization. JAOCS J. Am. Oil Chem. Soc..

[12] Hermund D.B. (2016). Extraction, Characterization and Application of Antioxidants from the Nordic Brown Alga Fucus vesiculosus. Kgs.

[13] Roohinejad S., Koubaa M., Barba F.J., Saljoughian S., Amid M., Greiner R. (2017). Application of seaweeds to develop new food products with enhanced shelf-life, quality and health-related beneficial properties. Food Res. Int..

[14] Cassani L., Gomez-Zavaglia A., Jimenez-Lopez C., Lourenço-Lopes C., Prieto M.A., Simal-Gandara J. (2020). Seaweed-based natural ingredients: Stability of phlorotannins during extraction, storage, passage through the gastrointestinal tract and potential incorporation into functional foods. Food Res. Int..

[15] Surendhiran D., Cui H., Lin L. (2019). Encapsulation of Phlorotannin in Alginate/PEO blended nanofibers to preserve chicken meat from Salmonella contaminations. Food Packag. Shelf Life.

[16] Sharifian S., Shabanpour B., Taheri A., Kordjazi M. (2019). Effect of phlorotannins on melanosis and quality changes of Pacific white shrimp (*Litopenaeus vannamei*) during iced storage. Food Chem..

[17] Ciko A.M., Jokić S., Šubarić D., Jerković I. (2018). Overview on the application of modern methods for the extraction of bioactive compounds from marine macroalgae. Mar. Drugs.

[18] Mekinić I.G., Skroza D., Šimat V., Hamed I., Čagalj M., Perković Z.P. (2019). Phenolic content of brown algae (Pheophyceae) species: Extraction, identification, and quantification. Biomolecules.

[19] Amarante S.J., Catarino M.D., Marçal C., Silva A.M.S., Ferreira R., Cardoso S.M. (2020). Microwave-Assisted Extraction of Phlorotannins from *Fucus vesiculosus*. Mar. Drugs.

[20] Vázquez-Rodríguez B., Gutiérrez-Uribe J.A., Antunes-Ricardo M., Santos-Zea L., Cruz-Suárez L.E. (2020). Ultrasound-assisted extraction of phlorotannins and polysaccharides from *Silvetia compressa* (Phaeophyceae). J. Appl. Phycol..

[21] Gall E.A., Lelchat F., Hupel M., Jégou C., Stiger-Pouvreau V. (2015). Extraction and purification of phlorotannins from brown algae. Methods Mol. Biol..

[22] Agregán R., Munekata P.E., Domínguez R., Carballo J., Franco D., Lorenzo J.M. (2017). Proximate composition, phenolic content and in vitro antioxidant activity of aqueous extracts of the seaweeds *Ascophyllum nodosum*, *Bifurcaria bifurcata* and *Fucus vesiculosus*. Effect of addition of the extracts on the oxidative stability of canola oil. Food Res. Int..

[23] Heffernan N., Brunton N., FitzGerald R., Smyth T. (2015). Profiling of the Molecular Weight and Structural Isomer Abundance of Macroalgae-Derived Phlorotannins. Mar. Drugs.

[24] Pinteus S., Silva J., Alves C., Horta A., Thomas O.P., Pedrosa R. (2017). Antioxidant and cytoprotective activities of fucus spiralis seaweed on a human cell in vitro model. Int. J. Mol. Sci..

[25] Jégou C., Kervarec N., Cérantola S., Bihannic I., Stiger-Pouvreau V. (2015). NMR use to quantify phlorotannins: The case of Cystoseira tamariscifolia, a phloroglucinol-producing brown macroalga in Brittany (France). Talanta.

[26] Stiger V., Deslandes E., Payri C.E. (2004). Phenolic contents of two brown algae, *Turbinaria ornata* and *Sargassum mangarevense* on Tahiti (French Polynesia): Interspecific, ontogenic and spatio-temporal variations. Bot. Mar..

[27] Ragan M.A., Jensen A. (1978). Quantitative studies on brown algae phenols. II. Seasonal Variation in polyphenol content of *Ascophyllum nodosum* (L.) Le Jol. and *Fucus vesiculosus* (L.). J. Exp. Mar. Biol. Ecol..

[28] Catarino M.D., Silva A.M.S., Mateus N., Cardoso S.M. (2019). Optimization of phlorotannins extraction from fucus vesiculosus and evaluation of their potential to prevent metabolic disorders. Mar. Drugs.

[29] Babbar N., Oberoi H.S., Sandhu S.K., Bhargav V.K. (2014). Influence of different solvents in extraction of phenolic compounds from vegetable residues and their evaluation as natural sources of antioxidants. J. Food Sci. Technol..

[30] Li Y., Fu X., Duan D., Liu X., Xu J., Gao X. (2017). Extraction and Identification of Phlorotannins from the Brown Alga, *Sargassum fusiforme* (Harvey) Setchell. Mar. Drugs.

[31] Francisco J., Horta A., Pedrosa R., Afonso C., Cardoso C., Bandarra N.M., Gil M.M. (2020). Bioacessibility of Antioxidants and Fatty Acids from *Fucus Spiralis*. Foods.

[32] Gager L., Connan S., Molla M., Couteau C., Arbona J.F., Coiffard L., Cérantola S., Stiger-Pouvreau V. (2020). Active phlorotannins from seven brown seaweeds commercially harvested in Brittany (France) detected by 1H NMR and in vitro assays: Temporal variation and potential valorization in cosmetic applications. J. Appl. Phycol..

[33] Ford L., Theodoridou K., Sheldrake G.N., Walsh P.J. (2019). A critical review of analytical methods used for the chemical characterisation and quantification of phlorotannin compounds in brown seaweeds. Phytochem. Anal..

[34] Koivikko R., Loponen J., Pihlaja K., Jormalainen V. (2007). High-performance liquid chromatographic analysis of phlorotannins from the brown alga *Fucus vesiculosus*. Phytochem. Anal..

[35] Olate-Gallegos C., Barriga A., Vergara C., Fredes C., García P., Giménez B., Robert P. (2019). Identification of Polyphenols from Chilean Brown Seaweeds Extracts by LC-DAD-ESI-MS/MS. J. Aquat. Food Prod. Technol..

[36] Steevensz A.J., MacKinnon S.L., Hankinson R., Craft C., Connan S., Stengel D.B., Melanson J.E. (2012). Profiling phlorotannins in brown macroalgae by liquid chromatography-high resolution mass spectrometry. Phytochem. Anal..

[37] Melanson J.E., Mackinnon S.L. (2015). Characterization of phlorotannins from brown algae by LC-HRMS. Methods Mol. Biol..

[38] Wang T., Jónsdóttir R., Liu H., Gu L., Kristinsson H.G., Raghavan S., Ólafsdóttir G. (2012). Antioxidant capacities of phlorotannins extracted from the brown algae *Fucus vesiculosus*. J. Agric. Food Chem..

[39] Agregán R., Munekata P., Franco D., Carballo J., Barba F., Lorenzo J. (2018). Antioxidant Potential of Extracts Obtained from Macro- (*Ascophyllum nodosum*, *Fucus vesiculosus* and *Bifurcaria bifurcata*) and Micro-Algae (*Chlorella vulgaris* and *Spirulina platensis*) Assisted by Ultrasound. Medicines.

[40] Tierney M.S., Soler-vila A., Croft A.K., Hayes M. (2013). Antioxidant Activity of the Brown Macroalgae *Fucus spiralis* Linnaeus Harvested from the West Coast of Ireland. Curr. Res. J. Biol. Sci..

[41] Hwang T.-L., Sung P.-J., Liaw C.-C. (2019). Development and Application of Herbal Medicine from Marine Origin.

[42] Liu H., Gu L. (2012). Phlorotannins from brown algae (*Fucus vesiculosus*) inhibited the formation of advanced glycation endproducts by scavenging reactive carbonyls. J. Agric. Food Chem..

[43] Wang T., Jónsdóttir R., Ólafsdóttir G. (2009). Total phenolic compounds, radical scavenging and metal chelation of extracts from Icelandic seaweeds. Food Chem..

[44] Yen G.C., Duh P.D., Tsai H.L. (2002). Antioxidant and pro-oxidant properties of ascorbic acid and gallic acid. Food Chem..

[45] Catarino M.D., Silva A.M.S., Cardoso S.M. (2017). Fucaceae: A source of bioactive phlorotannins. Int. J. Mol. Sci..

[46] Rajauria G., Foley B., Abu-Ghannam N. (2016). Identification and characterization of phenolic antioxidant compounds from brown Irish seaweed *Himanthalia elongata* using LC-DAD–ESI-MS/MS. Innov. Food Sci. Emerg. Technol..

[47] Rodriguez-Jasso R.M., Mussatto S.I., Pastrana L., Aguilar C.N., Teixeira J.A. (2014). Chemical composition and antioxidant activity of sulphated polysaccharides extracted from *Fucus vesiculosus* using different hydrothermal processes. Chem. Pap..

[48] Fellah F., Louaileche H., Dehbi-Zebboudj A., Touati N. (2017). Seasonal variations in the phenolic compound content and antioxidant activities of three selected species of seaweeds from Tiskerth islet, Bejaia, Algeria. J. Mater. Environ. Sci..

[49] Mendes M. (2017). Antioxidant Contents of *Fucus vesiculosus* L., in Response to Environmental Parameters. Master’s Dissertation.

[50] Mancuso F., Messina C., Santulli A., Laudicella V., Giommi C., Sarà G., Airoldi L. (2019). Influence of ambient temperature on the photosynthetic activity and phenolic content of the intertidal *Cystoseira compressa* along the Italian coastline. J. Appl. Phycol..

[51] Berger R., Malm T., Kautsky L. (2001). Two reproductive strategies in baltic *Fucus vesiculosus* (phaeophyceae). Eur. J. Phycol..

[52] Vandanjon L., Maureen D., Maya P., Philippe D., Valarie S.-P., Gilles B., Nathalie B. (2017). Seasonal Variation of *Sargassum Muticum* Biochemical Composition Determined by Fourier Transform Infra-Red Spectroscopy. J. Anal. Bioanal. Sep. Tech..

[53] Coelho S., Rijstenbil J.W., Sousa-Pinto I., Brown M.T. (2001). Cellular responses to elevated light levels in *Fucus spiralis* embryos during the first days after fertilization. Plant Cell Environ..

[54] Cruces E., Rojas-Lillo Y., Ramirez-Kushel E., Atala E., López-Alarcón C., Lissi E., Gómez I. (2016). Comparison of different techniques for the preservation and extraction of phlorotannins in the kelp *Lessonia spicata* (Phaeophyceae): Assays of DPPH, ORAC-PGR, and ORAC-FL as testing methods. J. Appl. Phycol..

[55] Zar J.H. (2010). Biostatistical Analysis.

[56] Finney D.J. (1971). Probit Analysis.

[57] Dinno A. (2015). Nonparametric pairwise multiple comparisons in independent groups using Dunn’s test. Stata J..

